# Metallothionein loss in cancer cells contributes to increased mutations through defective DNA repair and metabolic imbalance

**DOI:** 10.64898/2026.07.06.736843

**Published:** 2026-08-12

**Authors:** Mirna Mina-Abouda, Amy C Rees, Della Evans, Evan Villamor, George Fullbright, Heather R Ghent, Madison A Clark, Wendy Y Zhang, Isabel Koehler, Isabella Berry, Sydney Oesch, Robert Hutchinson, Davide Delisi, Christopher de Solis, Alexander Y Maslov, Christopher Bradley, Samim Sharifi, Rosemarie Elloisa P Acero, Yuri K Peterson, Jie Zhang, Zhiwei Ye, Tori C Rodrick, Danyelle M Townsend, Saverio Gentile, Brian Orr, Drew Jones, Jessica H Hartman, David T Long, Jonathan T Sczepanski, Joe R Delaney

## Abstract

Understanding which genes are involved in mutagenesis is essential for developing cancer prevention and treatment strategies; establishing protectors of the genome has revolutionized cancer biology. Here, we describe metallothionein (MT) proteins as previously uncharacterized protectors against mutagenesis. MT is a heavy metal binding protein essential for zinc homeostasis and protection against heavy metal cytotoxicity. Because zinc binds approximately 10-15% of the proteome and is critical for processes such as DNA repair and mitochondrial health, MT loss is expected to disrupt these processes. We hypothesized that MT loss induces genomic instability by impairing DNA repair and mitochondrial function. In this study, the consequences of MT deficiency in high-grade serous ovarian cancer (HGSC) were investigated by knockdown of the most highly expressed MT, *MT2A* . Loss of *MT2A* resulted in the impaired DNA repair pathway base excision repair (BER), leading to increased mutagenesis. *MT2A* deficiency produced mitochondrial dysfunction, characterized by a decrease in mitochondrial membrane potential, glycolysis, oxidative phosphorylation, amino acids, and an imbalance of nucleobases. Together, these defects reflect cellular states associated with increased cancer aggressiveness. These findings identify MT as a fundamental hub maintaining genomic and metabolic integrity.

**Abstract Figure:**

## Full Text Availability

The license terms selected by the author(s) for this preprint version do not permit archiving in PMC. The full text is available from the preprint server.

