# Supplementary material for: Metallothionein loss in cancer cells contributes to increased mutations through defective DNA repair and metabolic imbalance
Source: bioRxiv. 2026 Jul 7:2026.07.06.736843. Preprint. [Version 1] doi: 10.64898/2026.07.06.736843 (PMC13370963; doi:10.64898/2026.07.06.736843)

Supplementary Fig. S1: Mutational analysis in context of passaging in $CdCl_2$

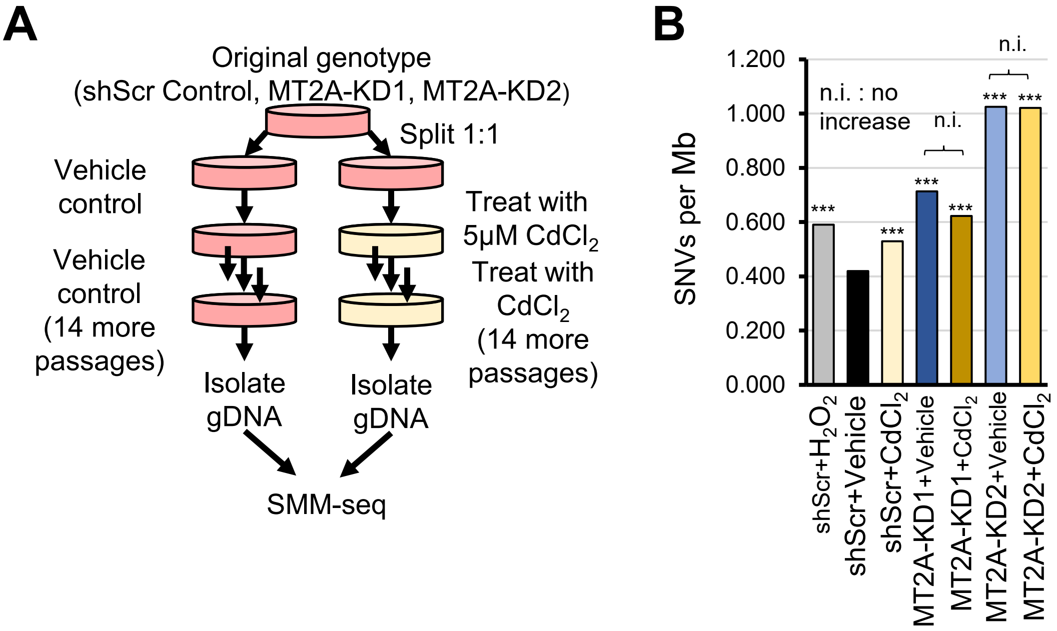

Supplementary Fig. S2: Dose-sensitive growth changes with MT2A-KD

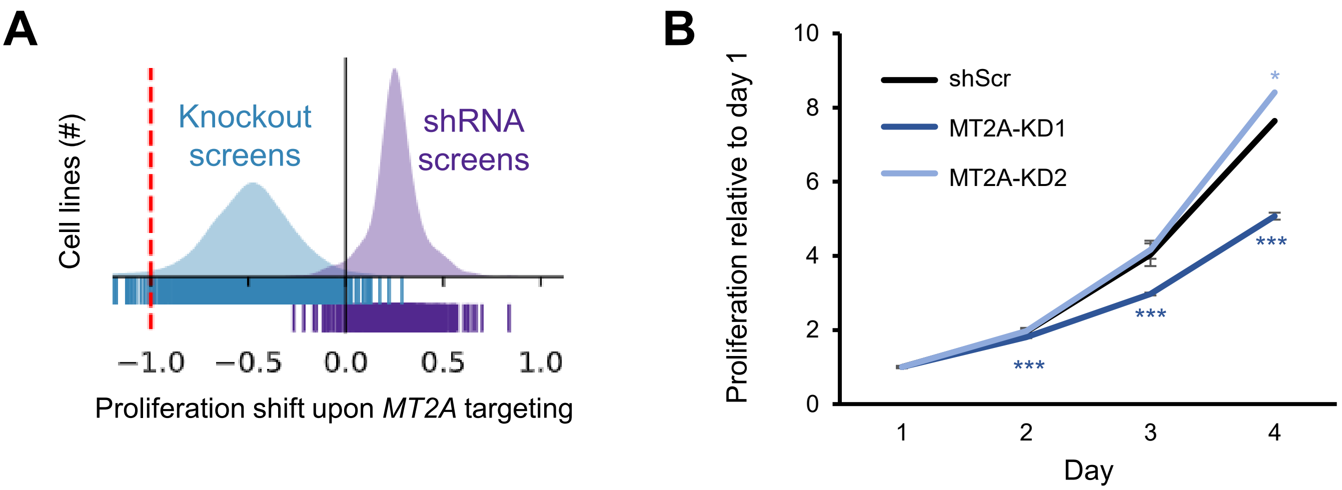

Supplementary Fig. S4:
Established SBS signatures cosine most-similar to *MT2A*-low SBS signatures

A MT2Alow SBS-ROS:SBS18 (Possibly damage by reactive oxygen species)
cosine similarity = 81.8%

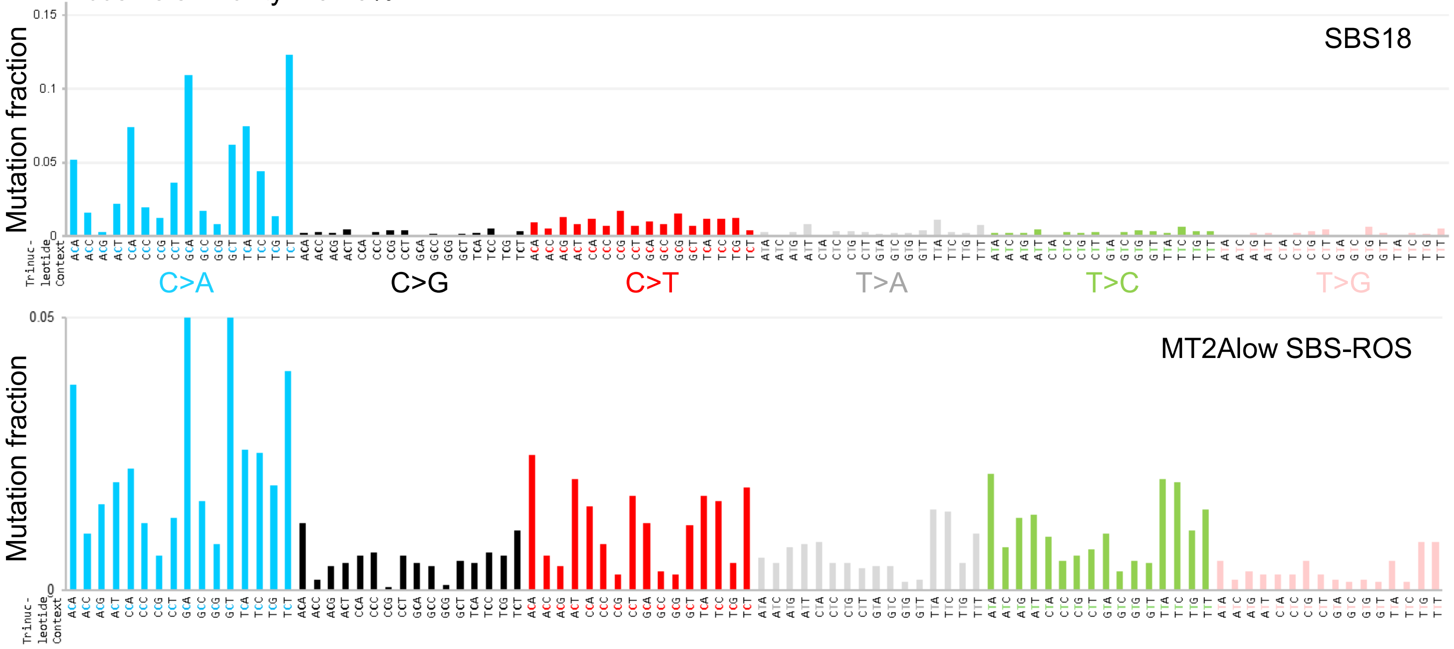

B MT2Alow SBS-BER:SBS30 (base excision repair due to inactivating mutations in *NTHL1*)
cosine similarity = 75.6%

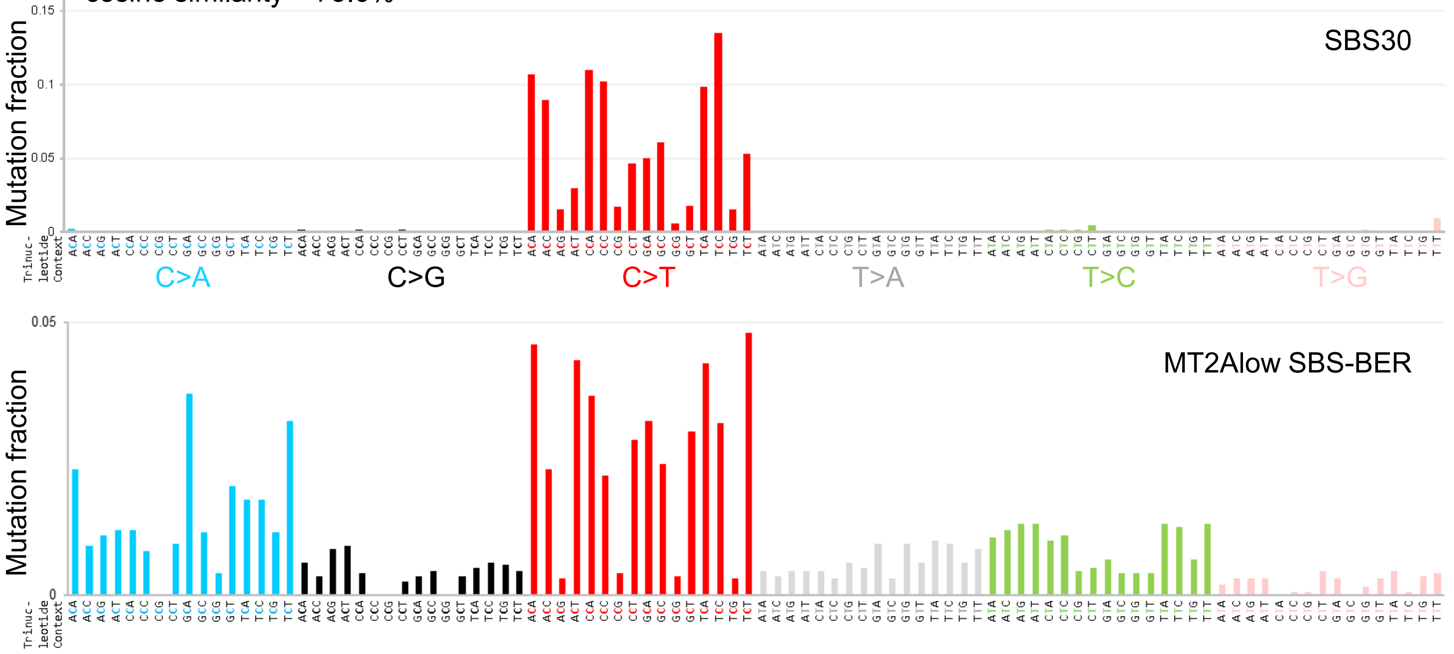

Supplementary Fig. S5:
Novel signature attributable mutations per cancer type by *MT2A* loss

MT2A-low *de-novo* (ROS-like) SBS signature MutationPatterns analysis of TCGA tumors
Colored: *MT2A* heterozygous loss
Gray: No *MT2A* loss

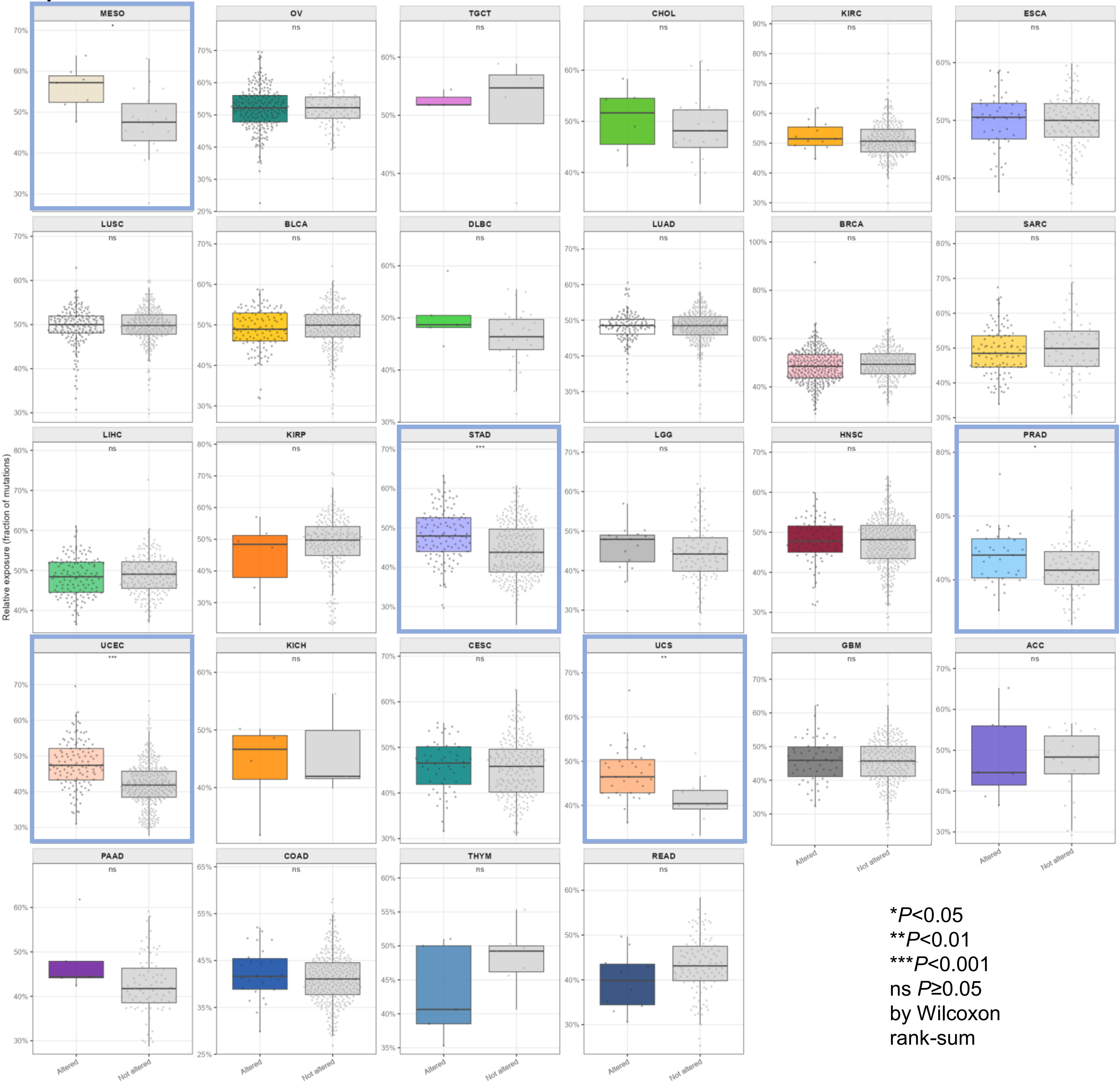

*P<0.05
**P<0.01
***P<0.001
ns P≥0.05
by Wilcoxon rank-sum

Supplementary Fig. S6: No 8-oxo-dG or MMS sensitivity changes with MT2A-KD

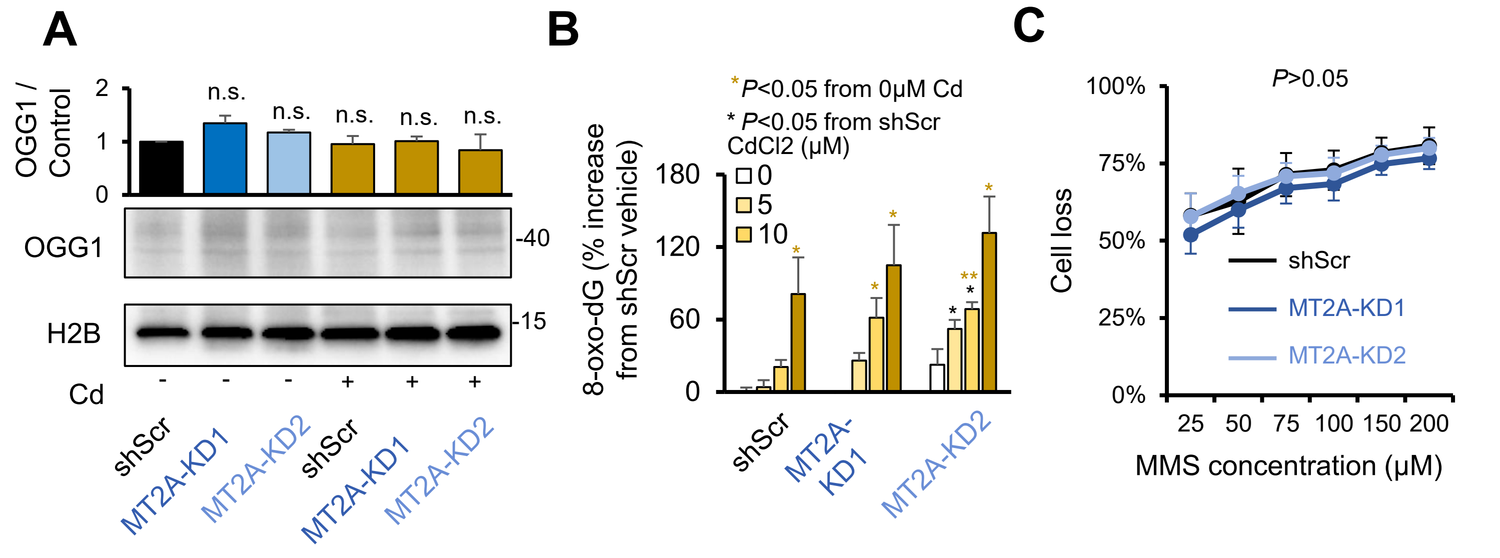

Supplementary Fig. S7: Scratch wound assay in MT2A-KD cells

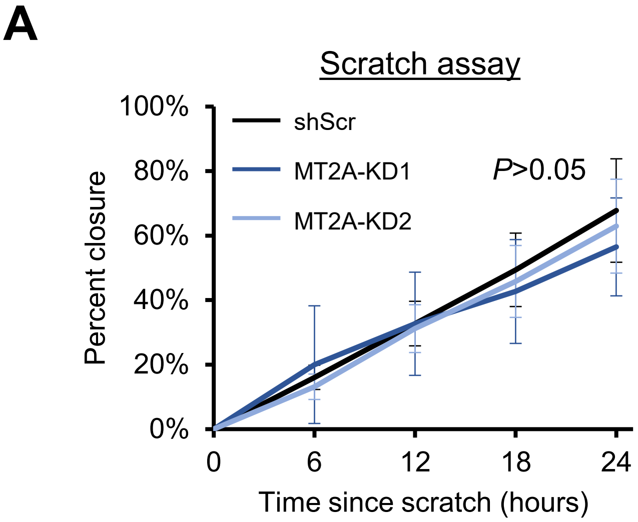

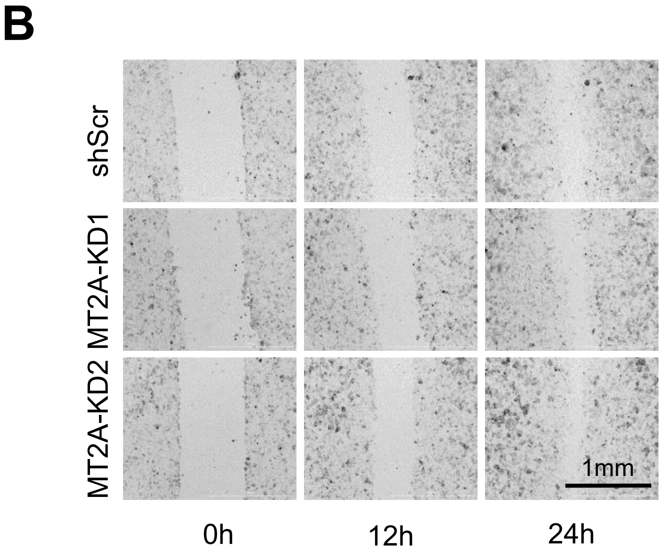

Supplementary Fig. S8: F318LOVi2 Mt2-KD metabolic changes

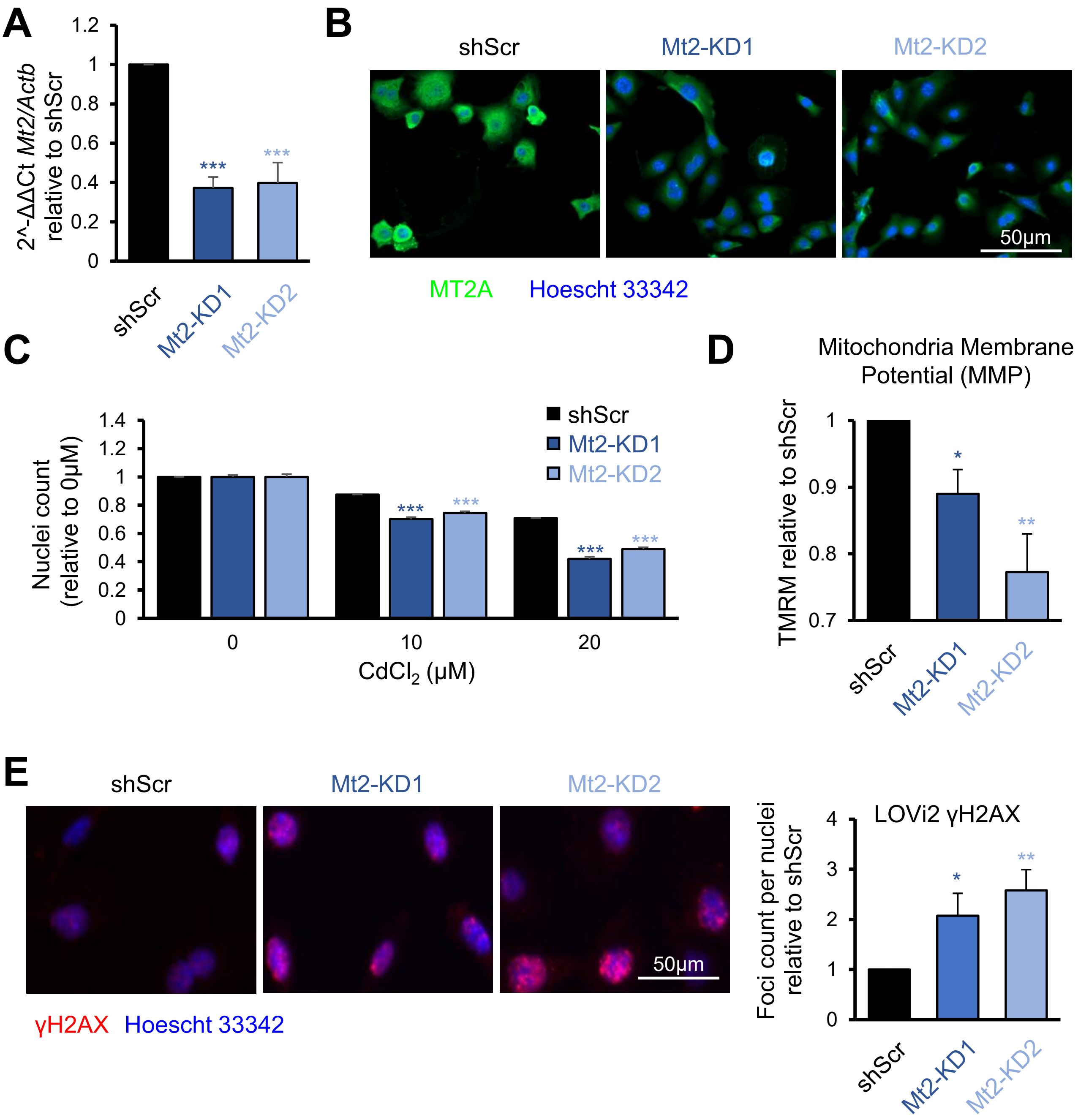

Supplementary Fig. S9: No peroxide sensitivity changes in MT2A-KD

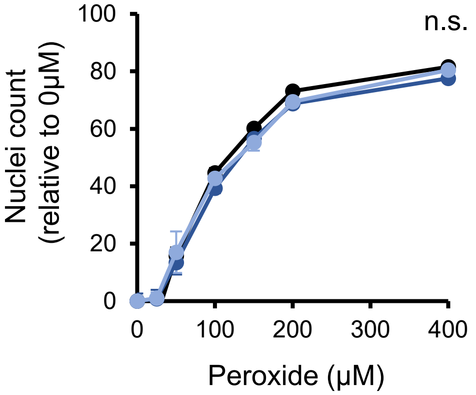

Supplement: Supplement 1 — Supplementary Figure S1. Mutational analysis in the context of passaging in CdCl2. (A) Schematic of genomic DNA sample collection for use in SMM-seq. (B) Quantitation of any SNV from SMM-seq data with indicated statistics derived from Fisher’s exact tests. Supplementary Figure S2. Dose-sensitive growth changes with MT2A-KD. (A) DepMap output of cancer cell line screens from RNAi screens and CRISPR-Cas9 knockout screens, with a null effect of a gene knockdown centered at 0. Positive numbers indicate faster growth whereas negative numbers indicate impaired growth and cancer dependency. (B) Proliferation assay of CAOV3 MT2A-KD cells compared to shScr. Supplementary Figure S3. Spontaneous single nucleotide variant analysis in the context of CdCl2. (A) Specific nucleotide changes SNVs per Mb of DNA analyzed by SMM-seq, including cadmium treatment. (B) SBS signatures as a fraction of total mutations, ranked by those most present in vehicle samples and compared to CdCl2 treated (excluding peroxide) samples. Supplementary Figure S4. Established SBS signatures cosine most-similar to MT2A-low SBS signatures. (A) Cosine similarity analysis of SBS MT2A-low-ROS indicated the highest similarity to SBS18, plotted in trinucleotide context here. (B) Cosine similarity analysis of SBS MT2A-low-BER indicated the highest similarity to SBS30, plotted in trinucleotide context here. Supplementary Figure S5. Novel signature attributable mutations per cancer type by MT2A loss. MutationalPatterns analysis of TCGA tumors for the MT2A-low (ROS-like) de-novo signature, indicating the percent of SNV mutations that may be attributable to the de-novo signature. Colored box plots indicate heterozygous MT2A loss, and gray represents no MT2A loss. Supplementary Figure S6. No 8-oxo-dG or MMS sensitivity changes with MT2A-KD. (A) Western blots from samples with indicated genetics and antibodies (mean +/− s.e.m. from 2 experiments). (B) Quantitation of an ELISA-based 8-oxo-dG assay (mean +/− s.e.m. from [file media-1.pdf]
